# Isolated atrial amyloidosis from atrial natriuretic peptide – a common but overlooked cardiac condition

**DOI:** 10.48101/ujms.v131.14458

**Published:** 2026-06-09

**Authors:** Per Westermark, Gerhard Wikström, Per Eldhagen

**Affiliations:** a Department of Immunology, Genetics and Pathology, Uppsala University, Uppsala, Sweden; b Department of Medical Sciences, Cardiology, Uppsala University, Uppsala, Sweden; c Department of Medicine, Karolinska Institute and Department of Cardiology, Karolinska University Hospital, Stockholm, Sweden

**Keywords:** Cardiac amyloidosis, localized amyloidosis, atrial fibrillation, cardiac insufficiency, fibrosis

## Abstract

Interest in the misfolding and aggregation of human proteins into amyloid fibrils has increased dramatically in recent years. The systemic amyloidoses constitute a group of serious disorders that often affect the heart. Each systemic amyloid type is derived from one of several plasma proteins, most commonly immunoglobulin light chains or transthyretin. Rapid progress has been made in developing effective treatments for several systemic forms.

There is also growing evidence that localized amyloids – such as those found in the brain or in the islets of Langerhans – or their smaller prefibrillar aggregates can exert toxic effects on nearby cells. However, other localized amyloids remain insufficiently understood. Isolated atrial amyloid (IAA) is derived from the polypeptide hormone atrial natriuretic peptide (ANP), which is expressed by atrial cardiomyocytes. IAA is a common age-related amyloidosis, affecting the left atrium more frequently than the right, with highly variable prevalence reported across studies.

The pathogenesis of IAA is unknown, although increased local concentrations of the precursor polypeptide are typically important in other hormone-derived amyloid disorders. IAA is suspected to contribute to the development of atrial fibrillation, but its potential mechanisms remain unclear and have been difficult to investigate due to the lack of methods for visualizing deposits in vivo. This paper, which provides an overview of the current research on IAA, highlights key gaps in knowledge and proposes approaches for studying IAA in vivo.

## Introduction

The presence of amyloid associated with aging in a variety of organs has been recognized for a very long time. Such deposits were often described in specific organs – such as the brain, the islets of Langerhans, or the heart – and were generally referred to as ‘senile amyloidosis’ (1). Before their biochemical nature could be determined, these deposits were frequently reported but attracted relatively little interest, as they were typically considered benign. There is, however, one important exception, wild-type transthyretin (ATTRwt) amyloidosis of the heart, which can lead to clinically significant cardiomyopathy.

The heart can be affected by several biochemical forms of amyloid deposits, both systemic and localized. It is a major target particularly in two biochemically distinct systemic diseases: amyloid immunoglobulin light chain (AL) amyloidosis, in which monoclonal immunoglobulin light chains aggregate into fibrils, and amyloid transthyretin (ATTR) amyloidosis, which occurs in two principal forms – either hereditary, caused by one of many missense mutations in the *TTR* gene, or as the wild-type variant.

An early description of pronounced cardiac amyloidosis involving the entire heart and associated with aging was from 1878 (2). This type of age-associated cardiac amyloidosis was for many years diagnosed only at autopsy. Smaller deposits confined to the atria were described much later. Because these atrial deposits were found to be more common than the more extensive amyloid infiltrating the ventricles, it was proposed that age-related cardiac amyloidosis begins in the atria and subsequently spreads to the rest of the heart (3, 4). However, only when it became possible to identify the biochemical classes of amyloid deposits and to distinguish them using specific analytical methods did it become clear that the amyloid localized to the atria is fundamentally different from the more widespread cardiac form (5). Therefore, the form with deposits throughout the entire heart was termed senile cardiac amyloidosis (SCA), whereas the form confined to the atria was designated isolated atrial amyloidosis (IAA) (5). However, although its clinical impact is primarily cardiac, it became evident that SCA is a systemic form of amyloidosis, with small deposits present in multiple organs. Consequently, the term ‘senile systemic amyloidosis’ (SSA) was introduced (6, 7). With the adoption of modern nomenclature based on the identity of the fibril protein, the designation SSA was replaced by the term ATTRwt (wild-type) amyloidosis (8).

### The cardiac atrium as an endocrine organ

The presence of granules in certain cardiomyocytes was first described by Kisch in 1956 (9) but their function was not understood until the discovery of atrial natriuretic peptide (ANP). Histochemical studies of the granules indicated a peptide composition containing methionine and tryptophan – residues now known to be present in proANP but not in the processed ANP peptide. Continued work by de Bold and colleagues demonstrated that extracts of heart tissue, particularly from the atria, possess natriuretic activity mediated through direct effects on the kidneys (10, 11). It was not until the discovery of ANP (11) that it became clear that the heart is, in fact, an endocrine organ containing stored peptide-hormone granules ready for release. Notably, the storage form is the inactive proANP, which differs from many other peptide hormones – such as insulin, whose storage form is predominantly the mature hormone. ANP is secreted in response to increased intra-atrial pressure and serves as an important regulator of blood volume and blood pressure. ANP exerts its effects by binding primarily to natriuretic peptide receptor 1 (NPR1, also known as NPRA).

### Atrial natriuretic peptide expression

**Figure 1:**
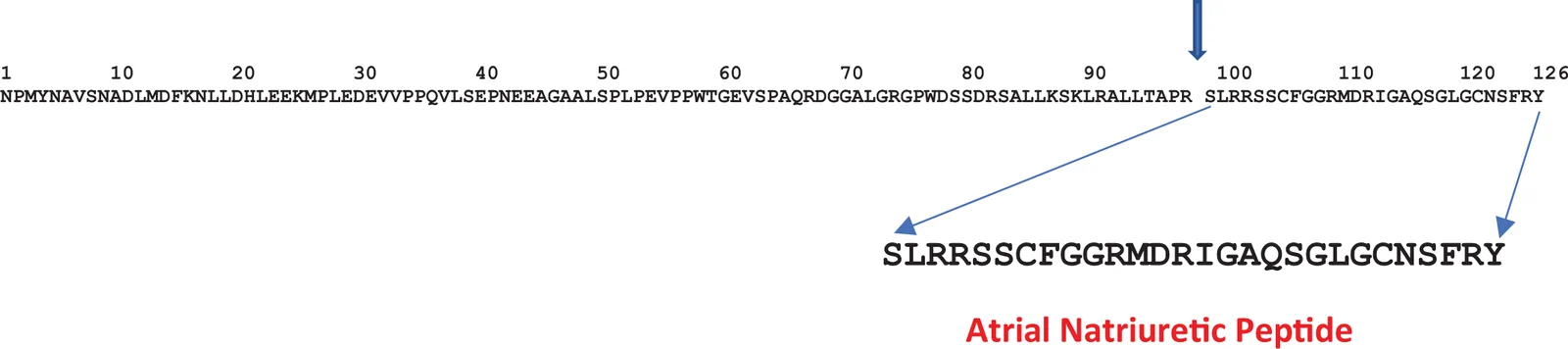
Human pro-atrial natriuretic peptide. ProANP is stored in atrial cardiomuscular granule and cleaved by corin at exocytosis, thereby releasing the 28 amino acid C-terminal ANP.

There are two related natriuretic peptides expressed by cardiomyocytes. ANP is almost exclusively synthesized in the cardiac atria, including the auricles, while the B-type natriuretic peptide (BNP) is produces by muscle cells in the ventricles (12) but also in the atria (13). ANP is expressed as a 151 amino acid proprotein in atrial cardiomyocytes. A 25 amino acid leader peptide is removed in the endoplasmic reticulum from which the 126 amino acid proANP (Figure 1) (14) is transferred to the Golgi apparatus where it is included in granule, which is the storage form of the hormone (15). At the exocytosis, proANP is cleaved into the active ANP molecule, constituting the 28 amino acid C-terminal part of the prohormone (Figure 1). The cleavage is performed by the transmembrane serine protease corin (16). Both ANP and the N-terminal fragment appear in the circulation (17).

The plasma concentration of ANP increases by up to ninefold with age in healthy humans (18). Plasma levels of ANP are markedly elevated in chronic congestive heart failure (19), most likely due to increased expression of the ANP precursor (20), although no increase in gene expression has been observed in patients with paroxysmal atrial fibrillation (21).

### Polypeptide hormone amyloidoses

The polypeptide hormone amyloidoses constitute a large group that includes ANP, islet amyloid polypeptide (IAPP), prolactin, parathyroid hormone (PTH), calcitonin, somatostatin, and glucagon (8, 22–24). In most cases, the amyloid fibrils are composed of the mature, full-length peptides, although precursor forms may also be involved (25).

### IAA fibril formation

The IAA fibrils are derived from ANP, first demonstrated by immunohistochemistry (26) and later confirmed through purification of the major fibrillar protein followed by amino acid sequence analysis (27, 28). Only the mature 28-amino-acid ANP was identified when the IAA protein was purified and sequenced (27, 28). Although the predominant component of IAA fibrils is the 28-amino-acid ANP, immunohistochemical studies have indicated the presence of small amounts of both proANP and brain-type natriuretic peptide together with ANP in IAA (29). Other regions of proANP, or even the entire precursor molecule, may represent minor constituents of the IAA fibrils (29, 30). A heptapeptide, KLRALLT, corresponding to residues 88–94 of proANP, has been shown to be highly amyloidogenic (31) in vitro and could potentially serve as an initiating segment for fibril formation if exposed. However, it is common for such amyloid-prone motifs to be buried within proteins, where they are shielded from aggregation (32, 33). An amyloidogenic protein must also be present above a critical concentration, beyond which nucleation can occur. In vivo, numerous factors interact – including pH, salt concentrations, and the presence of chaperones – which protect the cell from the formation of deleterious aggregates (34).

Amyloid fibril formation is a nucleation-dependent process, and the formation of a nucleus is likely stochastic. Once formed, the fibril acts as a catalyst, inducing the misfolding of adjacent native protein molecules, which are then incorporated at the fibril ends. Growth continues as long as the protein concentration remains above the critical threshold. The fibrils themselves are relatively stable and persist for long periods, whereas prefibrillar aggregates may appear only intermittently. This prion-like behavior accounts for the propagation of protein assemblies observed in various amyloid diseases (35, 36). The spread of amyloid depends on fibril fragmentation and the transport of smaller aggregates through various mechanisms – for example, via the bloodstream – to new deposition sites. Oligomeric aggregates, rather than fully formed fibrils, are thought to exert toxicity on nearby cells and are often considered more important than mature fibrils in driving tissue damage in several diseases, including Alzheimer’s disease, type 2 diabetes, and Parkinson’s disease (37–39). Whether such a mechanism exists in the ANP amyloid pathway is not known.

**Figure 2:**
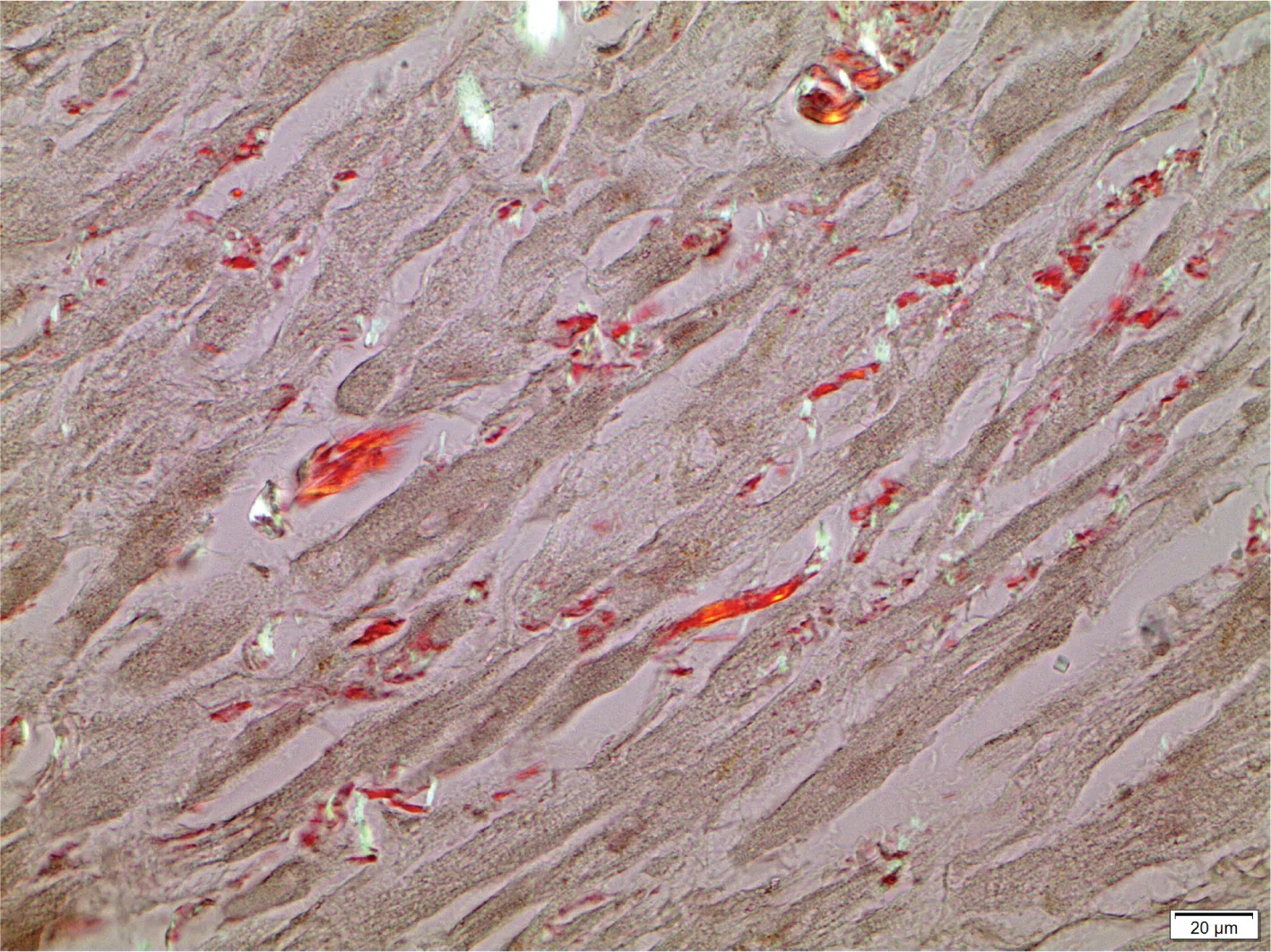
Characteristic appearance of IAA with streaks and dots of AANP amyloid but without larger deposits, typical of ATTRwt amyloidosis, shown in Figure 3. Section stained with Congo red and examined with partially crossed polarizers. Bar = 20 µm.

**Figure 3:**
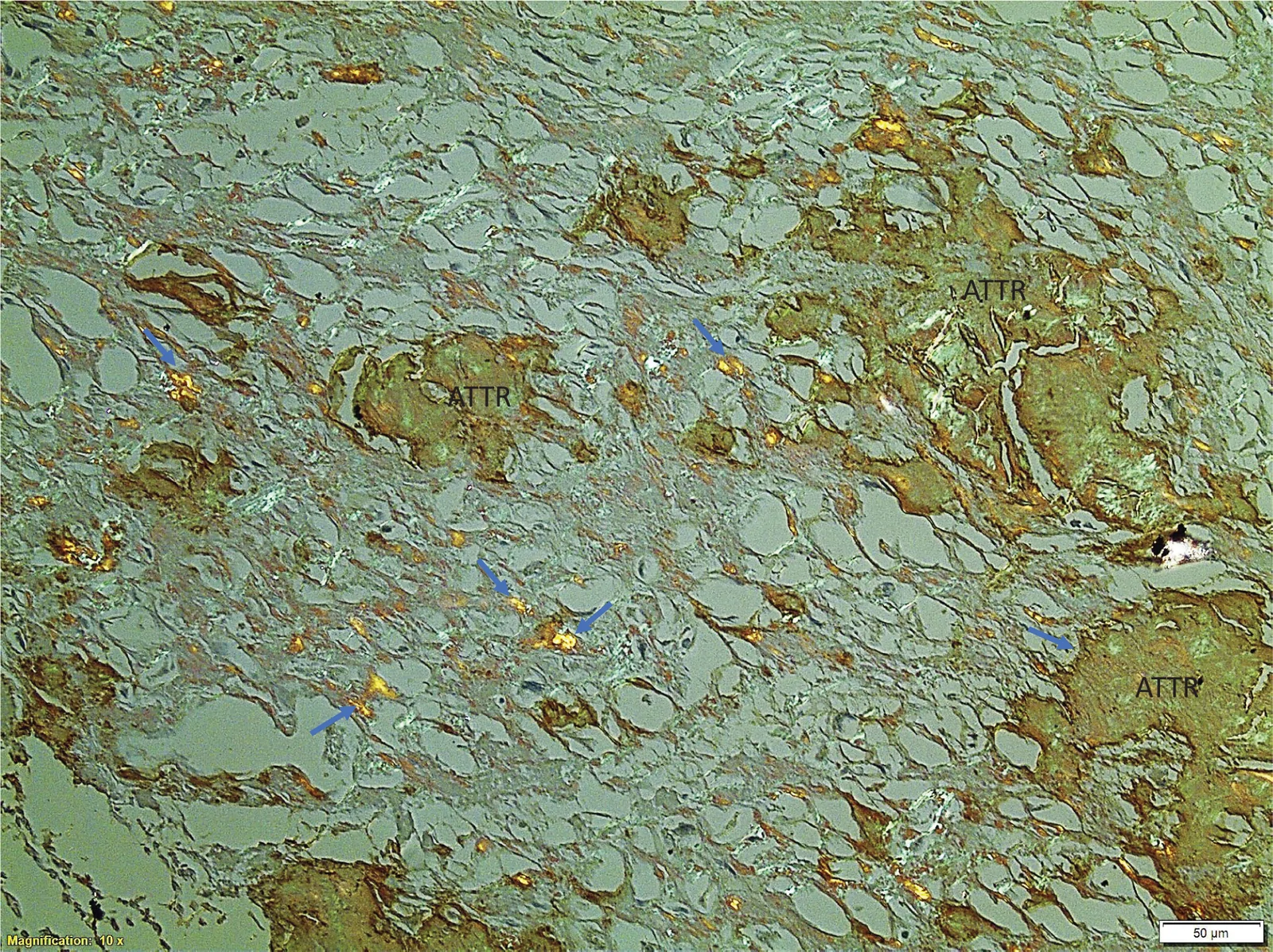
Two amyloid forms meet, ATTRwt and IAA (AANP). Multiple areas with ATTR amyloid (ATTR), immunolabeled for TTR. In addition, many small amyloid IAA deposits, not labeled with the antibody but recognized by staining with Congo red and showing typical birefringence in polarized light (blue arrows). Heart tissue, fixed in formalin and embedded in paraffin. Section immunolabeled with the monoclonal mouse antibody 7X, visualized with DAB and then double-stained with Congo red and examined in polarized light with crossed polarizers. Bar = 50 µm.

**Figure 4:**
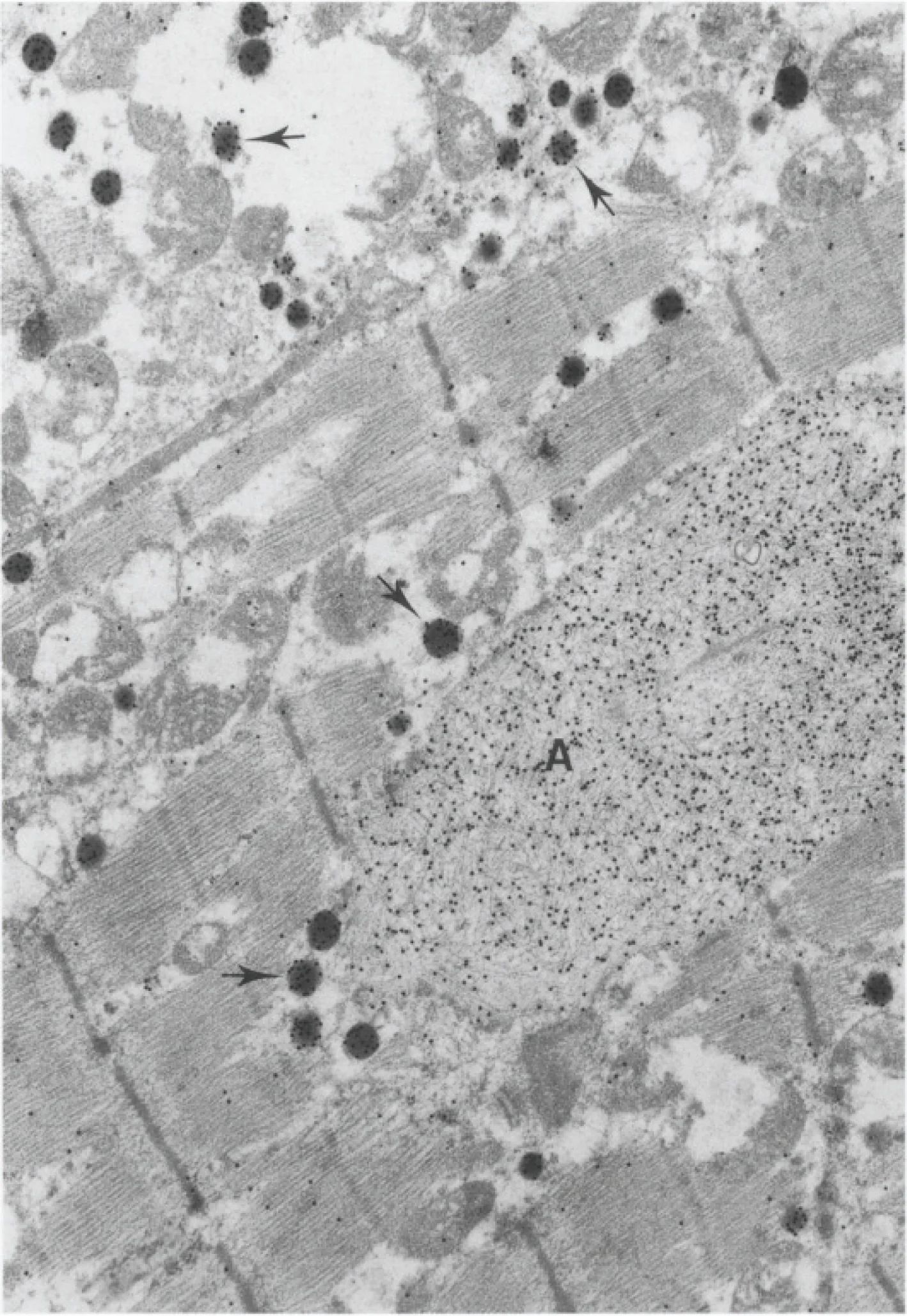
Electron microscopical image of an atrial cardiomyocyte with apparently intracellular bundles of amyloid fibrils (A). The section was immunogold labeled with antibodies against ANP and visualized with 10-nm gold particles. Figure from Johansson and Westermark (40), reproduced with permission.

IAA is a very common but underrecognized form of localized cardiac amyloidosis. It occurs in both atria, including the atrial appendages. IAA appears as thin extracellular streaks of congophilic material along individual atrial myocytes (Figure 2), but it may also involve small intramuscular blood vessels as well as the sub-intimal region. IAA (i.e. amyloid atrial natriuretic peptide [AANP] amyloid) is structurally distinct from deposits of ATTRwt amyloid – which can occur in the same tissue – in that ATTRwt deposits are more nodular and form discrete, compact amyloid areas (Figure 3). Although most IAA deposits are extracellular, small intracellular bundles are frequently observed within atrial cardiomyocytes (40) (Figure 4). IAA exhibits the characteristic features of amyloid, including its ultrastructural appearance on electron microscopy, its affinity for the dye Congo red, and the typical birefringence observed after Congo red staining (5, 40) (Figures 2 and 3).

It was shown early on that monomeric ANP readily forms typical amyloid fibrils in vitro (40) although recent studies have shown that an intact C-C ring is essential (41). Its cryoEM appearance has recently been described (41). The fibrils display two distinct polymorphs, both of which incorporate ANP dimers as their fundamental building blocks. In one polymorph, the dimers are arranged in an antiparallel orientation and are stabilized by a single disulfide bond (Cys7–Cys23). In the other, the ANP dimers are aligned in parallel and stabilized by two intermolecular disulfide bonds (Cys7–Cys7 and Cys23–Cys23). These findings indicate that dimerization plays a role in the formation of IAA fibrils, as previously suggested (42) although monomeric ANP readily forms fibrils in vitro (41, 42). It may be important to measure dimeric ANP specifically in order to understand its relevance in atrial pathology (41).

### IAA appearance and frequency

The prevalence of IAA in six different studies is presented in Table 1. Most of these studies are based on autopsy material, although a few derive from biopsy samples obtained during surgical procedures performed for various cardiac conditions. In the initial autopsy study in which IAA was first defined (5), a prevalence of 44 out of 72 hearts (61%) was found in unselected patients between 70 and 90 years of age. In another unselected autopsy series of 85 patients aged ≥80 years, IAA was present in 66 cases (78%) (43). Röcken et al. identified IAA in 40 of 245 right-atrial biopsies (16.3%) obtained during various cardiovascular surgical procedures. The patients ranged in age from 18 to 79 years (44). Steiner reported an age-dependent increase in prevalence, reaching 95% in individuals aged 81–90 years (45), while Looi (46) in a study of cardiac biopsies from 211 consecutive adult patients found deposits in 25 of 151 atrial appendages (16%). In contrast, Kawamura et al. reported IAA in 91 of 100 patients (91%) aged 65–89 years, although the inclusion criteria were not described in detail (47). Another study included 200 unselected autopsy cases, ranging from stillborn infants to individuals aged 90 years (45).

**Table 1:** Prevalence of IAA in six different studies.

Age span	Biopsy or autopsy	Total No. of patients	Amyloid in right atrium (%)	Amyloid in left atrium (%)	Amyloid in either atrium (%)	Combined with ATTR	Reference
70–99	Autopsy	72	-	-	44 (61)	5 (11)	(5)
80 -	Autopsy	85	61 (72)	64 (75)	64 (75)	19 (25)	(43)
60 -	Autopsy	100	-	-	30 (30)	2 (7)	(55)
0–90	Autopsy	200	-	-	103 (52)		(45)
10–60	Biopsy	71	6 (8)		-	-	(46)
10–60	Biopsy	80	-	19 (24)	-	-	(46)
65–89	Autopsy	100	63 (63)	82 (82)	91 (91)	0	(47)
24–79	Biopsy	245	40 (16)	-	-	4 (10)	(44)

ATTR: amyloid transthyretin.

Several factors may explain the variability in reported prevalence. The study materials were somewhat heterogeneous: some samples were obtained from both atria, whereas one major study (44) included tissue exclusively from the right atrium. The amount of amyloid was often limited, making IAA undetectable in hematoxylin–eosin staining. In most investigations, Congo red combined with polarization microscopy was used, which remains the standard method for demonstrating virtually all forms of amyloid. One report (44) employed Congo red fluorescence, and two others used Sirius red (45, 48) without polarization microscopy. In all studies except the first three (4, 43, 45), the ANP origin of the deposits was confirmed by immunohistochemistry. Despite the heterogeneity, the risk of confusing the two amyloid types (IAA and ATTRwt) is minimal because of their characteristic structural features in Congo red-stained sections (Figure 3). Other systemic amyloidoses are too rare to be considered.

The prevalence numbers vary quite remarkably but most studies share the finding that IAA increases in frequency with age, being rare before approximately 30–40 years of age (45, 46) after which the prevalence rises steadily. A higher frequency is generally observed in the left atrium compared with the right, including the auricles (46–48). IAA is also more common in females than in males (45, 47, 48).

Despite the high frequencies reported in prevalence studies, IAA is often described as a rare condition (49–53) or it is not considered at all (54), likely reflecting a general lack of awareness of the disorder. It is therefore not surprising that the simultaneous occurrence of IAA and ATTRwt amyloidosis is frequently reported as very uncommon. However, the co-occurrence was already noted in the original publication in which the two conditions were first defined (5) where it was found in 13 of 66 patients. Similar findings have been reported in other prevalence studies, including (43, 44, 55). A possible interaction between transthyretin (TTR) and ANP in the fibrillogenesis remains to be studied.

IAA and ATTR amyloidosis involving the atria and aortic valves without known ventricular involvement has been described, interestingly with positive ^99m^technetium-pyrophosphate scintigraphy imaging (56). It is most likely, however, that ATTRwt amyloidosis is always systemic, although deposits in many organs may be small despite being widely distributed (57).

### IAA and atrial fibrillation

There is no doubt that the substrate for amyloid fibril formation – ANP – is produced by atrial cardiomyocytes. It is likely, although not proven, that an increased local concentration of ANP contributes to amyloidogenesis, as has been suggested for other polypeptide hormone-producing tissues and tumors (22). It is also highly probable that amyloid, or its prefibrillar aggregates, affects the normal function of nearby cells, particularly atrial cardiomyocytes.

The possible association between IAA and atrial fibrillation has been examined in several studies. In one investigation based on right-atrial biopsy material, Röcken et al. found a significant association between both the presence and the extent of IAA and atrial fibrillation (44). Atrial fibrillation is a common consequence of various cardiovascular diseases, including valvular disorders, hypertension, coronary atherosclerosis, and heart failure. Low-voltage areas in the atria are frequently observed in persistent atrial fibrillation and are most often attributed to fibrosis (58, 59). The extent to which amyloid deposits contribute to atrial dysfunction and the appearance of low-voltage areas in atrial fibrillation remains insufficiently studied. Left atrial myocardial extensions to pulmonary veins (myocardial sleeves) are common and often harbor IAA although the importance in the pathogenesis of atrial fibrillation is uncertain (60).

A significantly increased prevalence of IAA has been reported in patients with congestive heart failure, particularly in the oldest age groups (61). However, it is likely that there was substantial overlap with concomitant atrial fibrillation in this cohort.

### Other possible clinical consequences of IAA

The impact of IAA on the atria is not known. In addition to atrial fibrillation, there are case reports in which IAA has been associated with other disorders. ATTRwt amyloidosis is associated with a risk of cardiac thrombosis with serious consequences, even in the absence of diagnosed atrial fibrillation (62). Whether IAA induces thrombus formation on its own or through its association with atrial fibrillation is unknown (44) Another very rare complication – intramural atrial hematoma – has been observed together with IAA in an otherwise healthy 40-year-old woman (63).

### Opportunities to study IAA in vivo

We have not identified any reports in which IAA deposits or their consequences have been studied in situ in living individuals. For such studies, it will be important to exclude patients with any degree of ATTR amyloidosis. It is not known whether calcified microdeposits occur in IAA, similar to those seen in type A ATTR amyloidosis (64), although a positive ^99m^Tc-diphosphono-propanodicarboxylic acid (^99m^Tc-DPD) scintigraphy has been reported in one case of IAA (65). Magnetic resonance imaging (MRI) combined with late gadolinium enhancement may be a potential approach, as it is used to assess atrial fibrosis (66), but a method to distinguish amyloid from connective tissue would be required. Fibrosis is also frequently associated with IAA, and both stain blue (though with different hues) using the commonly applied histologic stain for fibrosis, Masson’s trichrome. Both amyloid and fibrosis contribute to extracellular volume in ventricular cardiac biopsies in other forms of amyloidosis (67). It is likely that many published imaging and biopsy studies of atrial fibrosis include ANP amyloid within their reported values.

Theoretically, a positron emission tomography (PET) probe similar to Pittsburgh compound B (PIB) could be used to detect atrial amyloid. Biomarkers such as covalent ANP dimers in plasma – recently shown to constitute the IAA fibrils – may offer a new opportunity to link ANP to disease development (41). Still, current knowledge is very limited, and further clinical studies are needed.

In summary, despite IAA being one of the most prevalent localized amyloidoses and affecting the heart, its clinical significance remains largely unexplored.

## Notes on authors

All three authors have participated in the preparation of the manuscript and approved the submitted version.
